# Deep-subwavelength engineering of stealthy hyperuniformity

**DOI:** 10.1515/nanoph-2024-0541

**Published:** 2025-01-07

**Authors:** Jusung Park, Seungkyun Park, Kyuho Kim, Jeonghun Kwak, Sunkyu Yu, Namkyoo Park

**Affiliations:** Photonic Systems Laboratory, Department of Electrical and Computer Engineering, 26725 Seoul National University, Seoul 08826, Korea; Intelligent Wave Systems Laboratory, Department of Electrical and Computer Engineering, 26725 Seoul National University, Seoul 08826, Korea; Department of Electrical and Computer Engineering, Inter-University Semiconductor Research Center, and SOFT Foundry Institute, 26725 Seoul National University, 1, Gwanak-ro, Gwanak-gu, Seoul 08826, Korea

**Keywords:** disordered photonics, hyperuniformity, stealthy, localization, metamaterials, inverse design

## Abstract

Light behaviours in disordered materials have been of research interest primarily at length scales beyond or comparable to the wavelength of light, because order and disorder are often believed to be almost indistinguishable in the subwavelength regime according to effective medium theory (EMT). However, it was demonstrated that the breakdown of EMT occurs even at deep-subwavelength scales when interface phenomena, such as the Goos–Hänchen effect, dominate light flows. Here we develop the engineering of disordered multilayers at deep-subwavelength scales to achieve angle-selective manipulation of wave localization. To examine the disorder-dependent EMT breakdown, we classify the intermediate regime of microstructural phases between deep-subwavelength crystals and uncorrelated disorder through the concept of stealthy hyperuniformity (SHU). We devise material phase transitions from SHU to uncorrelated disorder for distinct angular responses of wave localization by tailoring the short-range and long-range order in SHU multilayers. The result paves the way to the realization of deep-subwavelength disordered metamaterials, bridging the fields of disordered photonics and metamaterials.

## 1 Introduction

Engineering disorder has provided multifaceted design freedom for optical devices [1]: bandgap engineering without crystalline order [2], [3], imaging of biological tissues [4], transitions between ballistic and diffusive transport [5], [6], deterministic control of random lasing [7], [8], and disorder-induced topological transitions [9]. Compared to researches on engineering structural disorder in other domains [10], [11], the uniqueness of engineering disorder in wave physics lies in utilizing wave interferences. Therefore, most studies have focused on the systems of which the characteristic lengths are beyond or comparable to the wavelength of light. At length scales below the wavelength, the phase evolution of a propagating light is negligible, which substantially degrades the impact of interferences. Therefore, a conventional way of modelling disordered materials at subwavelength scales is to introduce their effective media, rendering them almost indistinguishable from those of ordered materials.

On the other hand, the rise of subwavelength optics has transformed characteristic length scales in optics. When considerable phase evolutions occur at the interfaces through subwavelength geometry [12], the Goos–Hänchen effect [13], or non-Hermitian media [14], the necessary length scales for interferences are substantially reduced, as shown in metasurfaces [15], broken effective medium theory (EMT) in crystals [16], [17], and ultrathin resonances [18]. These achievements demonstrate that a light wave can sense subwavelength microstructures by exploiting interface physics, generalizing disordered photonics into subwavelength regimes. For example, wave localization traditionally studied in the wavelength-scale [19] was observed in multilayers with deep-subwavelength characteristic lengths [20], [21], [22]. The design freedom from subwavelength disorder also stimulates advanced functionalities in scattering from metasurfaces [23], [24], [25], [26], [27] and light outcoupling [28].

However, most researches in this emerging field have focused on the trivial transition from order to uncorrelated disorder, such as random perturbations without any correlations [20], [21], [23], [26], [28], or conducted the black-box-type analysis: numerical assessments to achieve target functionalities [22], [24], [27]. To fully exploit the abundant design freedom from engineered disorder, more in-depth studies unravelling the intricate relationships between multiple wave quantities and statistical features of subwavelength materials are highly desirable. It is thus timely to address the following question: “Can a light wave sense the correlation at deep-subwavelength scales?” Notably, recent studies on nonlocal theory for effective electromagnetic responses [29], [30], [31] underscore the need for further research, particularly in terms of engineering spatial correlations.

Here, we investigate the impact of engineering disorder at deep-subwavelength scales via analytical and numerical approaches, demonstrating that tailoring the pattern of disorder below *λ*/100 enables angle-selective manipulation of wave localization, even in the simplest geometry: one-dimensional (1D) multilayers. To examine nontrivial transitions between two extremes of material phases – crystals and uncorrelated disorder – we focus on stealthy hyperuniform (SHU) multilayers and their deformations in different length scales, which are achieved through the inverse design method based on the structure factor [32] and simulated annealing [33]. By analysing the EMT breakdown and angular responses of localization through the scattering matrix method [34], we demonstrate that the spatial correlation plays a critical role even in the deep-subwavelength regime, enabling angle-selective manipulation of optical transparency through disorder engineering. The result generalizes SHU in relation to wave behaviours at the interfaces, such as the Goos–Hänchen effect, extends the application range of the Born approximation, and provides indispensable functionalities for high-precision sensing [35], light outcoupling [28], and radiative cooling [36].

## 2 Results

### 2.1 Deep-subwavelength engineering of multilayers

As the simplest example of deep-subwavelength engineered disorder, we investigate a one-dimensional (1D) multilayer composed of two material phases with high and low refractive indices denoted as *n*_H_ and *n*_L_, respectively, as *n*_H_ > *n*_L_. The multilayer is surrounded by a homogeneous material with a refractive index *n*_ext_, and we assume the transverse electric (TE) planewave incidence from the surrounding material. To examine the impact of deep-subwavelength microstructures, we focus on a set of multilayers characterized by the identical effective refractive index *n*_EMT_, defined as *n*_EMT_ = [*f*_H_ *n*_H_^2^ + *f*_L_ *n*_L_^2^]^1/2^, where *f*_H_ and *f*_L_ denote the volume fractions of high- and low-index materials, respectively, both set at *f*_H_ = *f*_L_ = 0.5. It is worth mentioning that multilayers in this material set are indistinguishable with identical wave responses according to EMT [37], although it has already been disproven at two extremes – crystals [16], [21] and uncorrelated disorder [20], [21] – of microstructural phases, even under deep-subwavelength conditions.

In classifying materials regarding their wave properties, it is critical to employ suitable statistical parameters that properly extract the microstructural information [38], such as the perturbation strength, orders of correlations, and the clustering of constituents. In our study, we utilize the structure factor *S*(*k*) – the reciprocal-space representation of the two-point probability function [38], [39] (Supplementary Note S1 for the calculation of structure factors). Although *S*(*k*) directly determines light scattering under the first-order Born approximation, we extend our discussion beyond this approximation, as addressed later.

**Figure 1:**
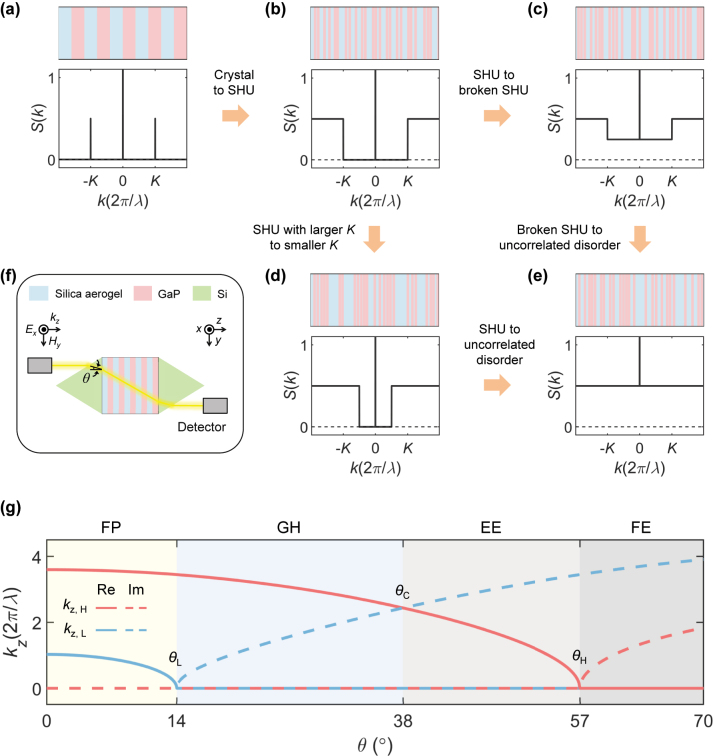
Deep-subwavelength engineered disorder. (a–e) Schematics for 1D multilayers with different degrees of disorder: crystal (a), SHU with larger *K* (b), deformed SHU with broken long-range order (c), SHU with smaller *K* (d), and Poisson uncorrelated disorder (e). The top and bottom subfigures in (a–e) illustrate the schematics of exemplified microstructures and their averaged *S*(*k*), respectively. The flow of the orange arrows represents the order-to-disorder transitions of interest in this work. (f) Schematic of the proposed experimental setup for measuring angular transmission. In the proposed setup, a silicon prism is employed to implement the critical angle condition in the excitation and modify the incident direction of an *E*_*x*_-polarized light to the multilayer. The light then passes through the multilayer and exits the prism for detection of transparency. (g) Classification of angular responses: FP (*θ* < *θ*_L_), GH (*θ*_L_ ≤ *θ* < *θ*_C_), EE (*θ*_C_ ≤ *θ* < *θ*_H_), and FE (*θ*_H_ ≤ *θ*) regimes. In (g), we assume *n*_H_ = 3.6, *n*_L_ = 1.03, and *n*_ext_ = 4.3, which lead to *θ*_L_ = 14°, *θ*_C_ = 38°, and *θ*_H_ = 57°. *k*_*z*,H_ and *k*_*z*,L_ denote the wavenumbers along the *z*-axis in high- and low-index materials, respectively. The details of materials for practical implementation are discussed in Section 4.

Despite the simplicity of multilayers considered, *S*(*k*) offers various ways of exploring microstructural phases (Figure 1a–e). At one end of the microstructural phase diagram, a 1D crystal is illustrated by the Bragg peaks [40] at *S*(*k*) (Figure 1a). At the opposite end, there is the Poisson uncorrelated disorder [38], which possesses the constant *S*(*k*) in the thermodynamic limit (Figure 1e). To explore the intermediate phases between these two extremes, we employ the concept of SHU [1], [29], [30], [31], [39], [41], [42], the suppression of long-wavelength density fluctuation as *S*(|*k*| < *K*) ≈ 0 for a specific positive value *K*. Notably, the SHU material exhibits the length-scale dependent degree of disorder: the crystal-like long-range (or small |*k*|) order and the Poisson-like short-range (or large |*k*|) order in terms of *S*(*k*) (Figure 1b). Therefore, we can envisage engineering disorder in terms of two distinct transitions from crystals to the Poisson disorder: degraded long-range order with broken SHU (Figure 1c) and degraded short-range order while maintaining SHU (Figure 1d).

To investigate the EMT breakdown in relation to phase evolutions across the interfaces, we examine oblique incidence at angle *θ* to engineered multilayers (Figure 1f), which allows for angle-dependent combinations of propagating and evanescent modes within a multilayer owing to the distinct critical angle of each material phase *n*_H_ and *n*_L_ (Figure 1g). Specifically, when *n*_ext_ > *n*_H,L_, three distinct angles characterize the wave properties of multilayers: *θ*_H_ for the critical angle from *n*_ext_ to *n*_H_, *θ*_L_ for the critical angle from *n*_ext_ to *n*_L_, and *θ*_C_ for the critical angle from *n*_ext_ to *n*_EMT_. Given the relationship *θ*_L_ < *θ*_C_ < *θ*_H_, the angular responses of *θ* are classified into four regimes [20] (Figure 1g): the fully propagating (FP) regime (*θ* < *θ*_L_), the Goos–Hänchen (GH) regime (*θ*_L_ ≤ *θ* < *θ*_C_), the EMT evanescent (EE) regime (*θ*_C_ ≤ *θ* < *θ*_H_), and the fully evanescent (FE) regime (*θ*_H_ ≤ *θ*). We explore wave behaviours in each angular regime in engineered disorder, extending beyond previous studies on crystals [16] and uncorrelated disorder [20], [21].

### 2.2 Deep-subwavelength SHU

As the first step of exploring the intermediate regime in microstructural phases, we investigate the uniqueness of the SHU multilayers compared to crystals and uncorrelated disorder. For the incidence at a free-space wavelength of *λ* = 500 nm, we analyse the wave localization properties of multilayers with thickness *L* across various material microstructures. The localization of each multilayer is quantified by the localization length *ξ*, which is defined statistically for disordered materials [43], as follows:

(1) $$\xi =-\frac{L}{\left\langle \log T\left(\theta \right)\right\rangle },$$

where *T*(*θ*) denotes the incident-angle-dependent transmission through a realization of multilayers calculated from the scattering matrix method [34], and ⟨…⟩ represents the ensemble average for random realizations of multilayers to examine the thermodynamic limit of disordered materials under the ergodic condition [38]. We focus on the angular range near the GH regime from *θ*_L_ = 14° to *θ*_C_ = 38°, where the substantial breakdown of EMT was observed in crystals [16] and uncorrelated disorder [20], [21].

**Figure 2:**
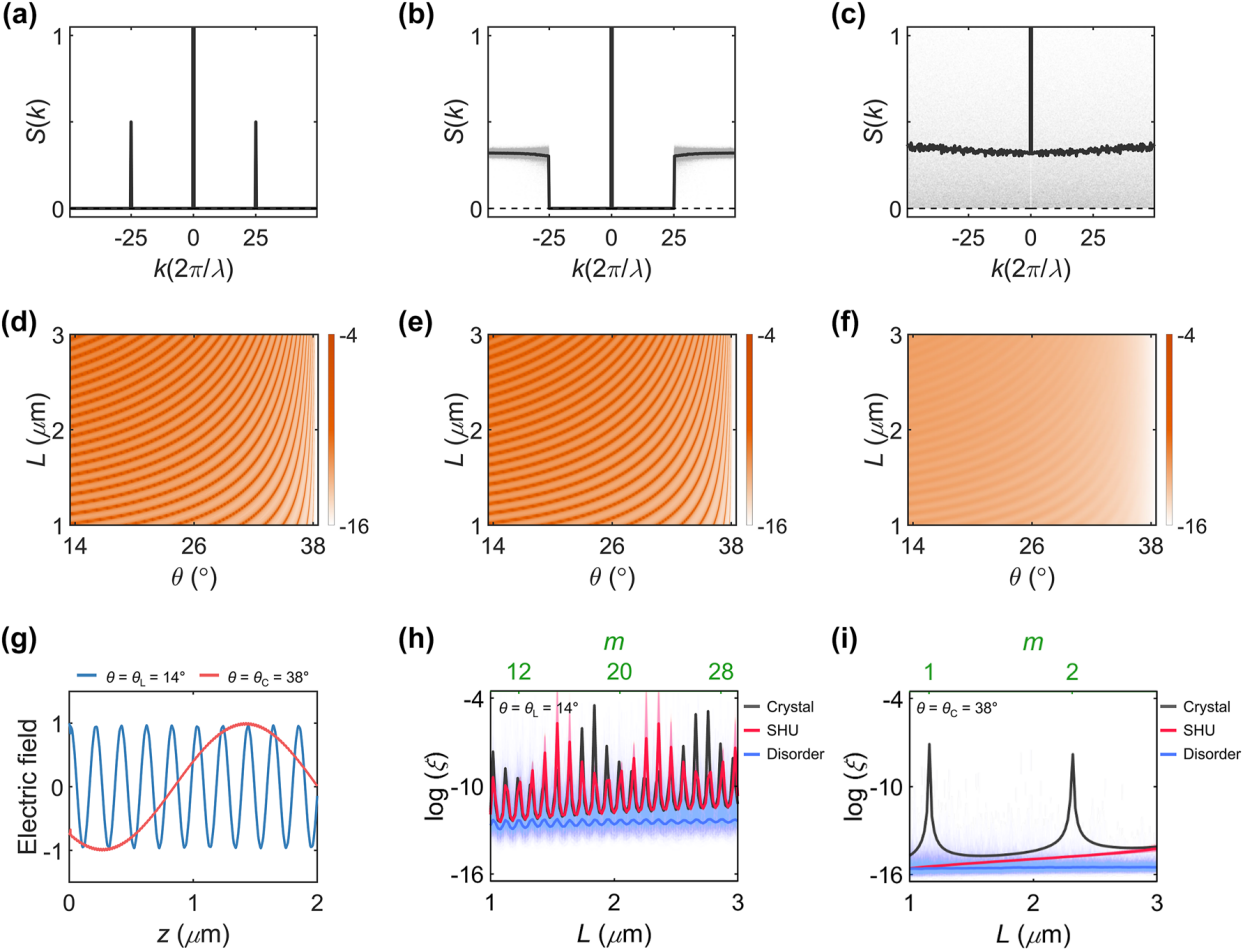
Deep-subwavelength SHU. (a–c) *S*(*k*) and (d–f) log(*ξ*(*θ*, *L*)) for a crystal (a, d), SHU (b, e), and uncorrelated disorder (c, f). *L* = 4*λ* and *K* = 50*π*/*λ* in (a–c). Black points and lines represent each realization and ensemble averages, respectively, in (a–c). (g) Normalized electric field amplitude along the *x*-axis within a crystal of *L* = 4*λ* for *θ* = *θ*_L_ = 14° and *θ* = *θ*_C_ = 38°. (h, i) Logarithmic plots of the localization lengths *ξ* as a function of *L* for *θ* = *θ*_L_ = 14° (h) and *θ* = *θ*_C_ = 38° (i). The upper horizontal axes in (h, i) represent the order *m* of the Fabry–Pérot resonance, which is determined by the Bloch wavenumber of a crystal having 20 nm periodicity. The fluctuation for crystals in (h) originates from the discretized sampling of *L* with crystal periodicity of 20 nm. Transparent and solid lines denote each realization and their ensemble averages, respectively. In analyzing SHU (b, e, h) and uncorrelated disorder (c, f, i), ensembles of 10^3^ realizations are examined. All the other parameters are the same as those in Figure 1.

Figure 2a–c shows the structure factors *S*(*k*) of a crystal and the statistically designed SHU and Poisson uncorrelated disorder, which are calculated for the multilayers of the thickness from *L* = 2*λ* to *L* = 6*λ*. While the crystal has a periodicity of 20 nm with the first-order Bragg peaks at |*k*| = *K* = 50*π*/*λ* (Figure 2a), the thickness of the high-index (*n*_H_) layers in both the SHU and uncorrelated disorder is set to 2 nm. These layers are iteratively placed in a perturbative manner inside the low-index (*n*_L_) background to achieve the target *S*(*k*), while maintaining *f*_H_ = *f*_L_ = 0.5 (see Section 4 and Supplementary Note S2 for the inverse design process). The design process successfully provides the target structure factors illustrated in Figure 1a–e; the SHU satisfies *S*(|*k*| < *K*) ≈ 0 and *S*(|*k*| ≥ *K*) ≈ *S*_0_ (Figure 2b), while *S*(|*k*| > 0) ≈ *S*_0_ in uncorrelated disorder (Figure 2c), where *S*_0_ is the constant determined by the averaged *S*(*k*) in uncorrelated disorder.

Localization lengths with respect to incident angles are shown in Figure 2d–f for each microstructural phase. Under the EMT, all the given deep-subwavelength multilayers are modelled as a homogeneous layer with the same effective index, *n*_EMT_ = [(*n*_H_^2^ + *n*_L_^2^)/2]^1/2^. This modelling leads to angular transmissions that exhibit Fabry–Perot resonance patterns (Figure 2d–f), where the effective wavelength is determined by the incident angles (Figure 2g). However, although the identical *ξ*(*θ*, *L*) regardless of microstructures is expected in the EMT modelling, the EMT breakdown leads to the substantial decrease of *ξ*(*θ*, *L*) in uncorrelated disorder (Figure 2f) compared to that of crystals (Figure 2d).

The importance of correlation in a deep-subwavelength regime is clearly demonstrated by the localization property of SHU multilayers. Although the overall localization behaviours of the SHU look very similar to those of a crystal (Figure 2d and e), a thorough analysis of the incident angle *θ* unveils the uniqueness of SHU (Figure 2h and i). Near the boundary between the FP and GH regimes (*θ* ≈ *θ*_L_ = 14°, Figure 2h), the evolution of localization around varying thicknesses shows similarities in both a crystal and the SHU in terms of the widths and peak positions of *ξ*(*L*). However, when the effect of the Goos–Hänchen interface phase shifts becomes more pronounced, which occurs near the boundary between the GH and EE regimes (*θ* ≈ *θ*_C_ = 38°, Figure 2i), localization behaviours in the SHU no longer follow those in a crystal, and instead, resemble the broadening shown in uncorrelated disorder. This result reveals that the length-scale-dependent order of the SHU – characterized by crystal-like long-range order and Poisson-like short-range order – directly affects its angular responses in the GH regime – exhibiting crystal-like localization near the FP regime and Poisson-like localization near the EE regime. Notably, because the discrepancy between the FP and EE regimes stems from the presence of evanescent modes inside multilayers (Figure 1g), the uniqueness in the angular responses of each microstructure – a crystal, SHU, and uncorrelated disorder – originates from the distinct distributions of evanescent modes. Therefore, we can envisage more selective and deterministic engineering of optical angular functionalities near the EE regime, by independently manipulating short- and long-range order and the corresponding evanescent-mode distributions.

### 2.3 Nontrivial transitions from SHU to uncorrelated disorder

**Figure 3:**
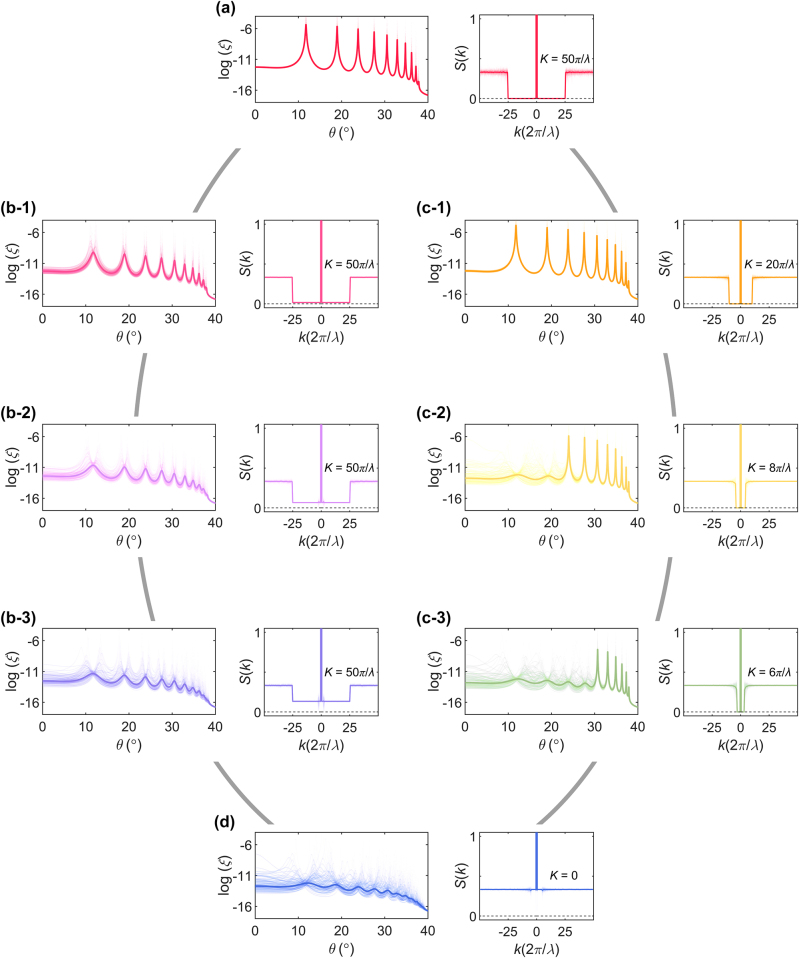
Transitions across different degrees of disorder through differentiated tailoring of length-scale order. (a–d) Localization lengths and the corresponding structure factors as functions of the incident angle *θ*: SHU (a), breakdown of long-range order (b: b-1, b-2, and b-3), decrease of *K* (c: c-1, c-2, and c-3), and uncorrelated disorder (d). In the left panels of (a–d), points and lines represent the realizations of log(*ξ*) and their ensemble averages log(⟨*ξ*⟩), respectively, where ⟨*ξ*⟩ denotes the average of each realization of *ξ*. In the right panels of (a–d), partially transparent and solid lines for *S*(*k*) represent each realization and ensemble averages, respectively. In all cases, ensembles of 10^2^ realizations are examined. We set the entire thickness as *L* = 2*λ* for the free-space wavelength *λ* = 500 nm, same as Figure 1. All the other parameters are the same as those in Figure 1.

To employ the concept of engineered disorder [1] to impose optical functionalities on deep-subwavelength microstructures, we investigate microstructural phase transitions between the SHU and uncorrelated disorder and their impact on localization (Figure 3). When we apply the inclusion of uncorrelated perturbations to the SHU state, the value of *S*(*k*) increases across the entire range of *k*, leading to the trivial transition from SHU to uncorrelated disorder and the consequent gradual changes in wave behaviours. Instead of such a trivial transition, we devise the nontrivial transitions that are clearly differentiated by their short-range and long-range order, as well as by the maintenance of SHU. The first transition is characterized by increasing *S*(|*k*| < *K*), which corresponds to the breakdown of long-range order (Figure 3a, b, and d). At the same time, the multilayers are no longer SHU because *S*(|*k*| < *K*) ≠ 0. In contrast, the second transition is designed with the decrease of *K*, which degrades the short-range order of multilayers, while maintaining their SHU with *S*(|*k*| < *K*) ≈ 0 (Figure 3a, c, and d). Notably, the range of engineered length scales maintains far below the wavelength of light: controlling *S*(*k*) below *λ*/25 in Figure 3b and across *λ*/10 (Figure 3c-1), *λ*/4 (Figure 3c-2), and *λ*/3 (Figure 3c-3). Examples of designed multilayer patterns are shown in Supplementary Note S3.

Figure 3 illustrates that these two nontrivial transitions deliver distinct evolutions in maintaining Fabry–Perot resonances. At the initial SHU state (Figure 3a), we observe distinct Fabry–Perot resonances as already depicted in Figure 2b, while the peak positions and angular widths vary across the FP, GH, and EE regimes due to changes in effective wavelengths. Through the first-type transition with increasing *S*(|*k*| < *K*), the breakdown of SHU leads to a highly sensitive annihilation of Fabry–Perot resonances, with the resonance peaks decreasing by an order of magnitude from a minor perturbation level of *S*(|*k*| < *K*) ≈ *S*_0_/20 for *S*(|*k*| ≥ *K*) ≈ *S*_0_ (from Figure 3a and b).

In contrast, the second-type transition, which involves decreasing *K* while maintaining SHU, enables the engineering of the angular range where Fabry–Perot resonances are preserved. Notably, an abrupt change in the Fabry–Perot resonances begins to occur near the SHU state at *K* = 10*π*/*λ* (Figure 3c-2 and c-3), initiating the annihilation of the highest-order of resonance due to disrupted short-range order. The further decrease in *K* sequentially annihilates lower-order resonances until the annihilation of the zeroth-order resonance as shown in Figure 3d.

### 2.4 Angle-selective engineering of localization

**Figure 4:**
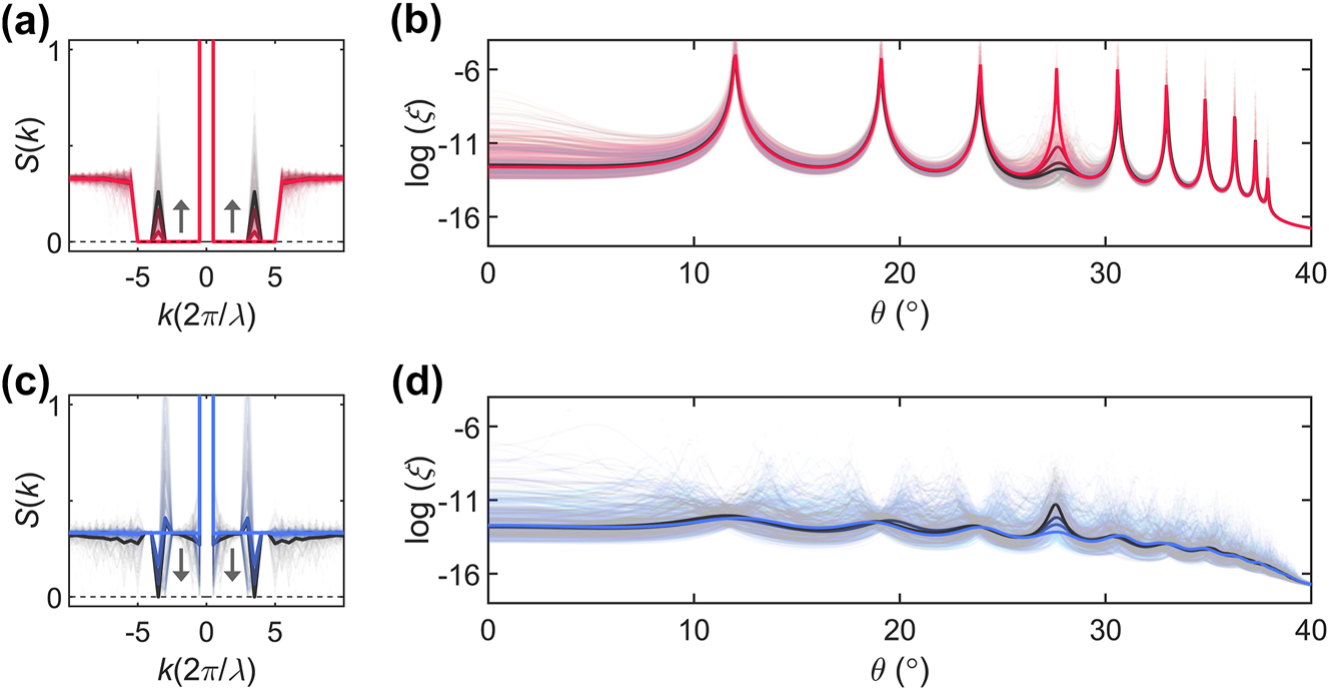
Angular-selective localization. (a, b) Selective annihilation and (c, d) selective creation of the target Fabry–Perot resonances: the designed *S*(*k*) transitions (a, c) and incident-angle-dependent localization lengths (b, d). In (a, c), the black arrows illustrate the transition for each case. All the other parameters and plots are the same as those in Figure 3.

Based on the results shown in Figure 3, which demonstrate the strong connection between the density fluctuation characterized by *S*(*k*) and wave localization, we further implement angle-selective engineering of localization. We employ the transition of material phases at a target length scale, focusing on initial states of SHU and uncorrelated disorder. Considering the angular range of interest depicted in Figure 3, we explore the manipulations of the SHU state near *K* = 10*π*/*λ*. Initially, the SHU multilayers exhibit Fabry–Perot resonances (Figure 4a and b, red lines), while uncorrelated multilayers show angularly flattened strong localization (Figure 4c and d, blue lines). To manipulate the density fluctuation at a specific length scale, we design an ensemble of disorder realizations generated by the structure factors *S*(*k*) of which the value at the target *k* increases (black line in Figure 4a) or decreases (black line in Figure 4c).

Through this length-scale-specific engineering of deep-subwavelength microstructures, the resonance at a specific angle can be selectively controlled over a few orders of magnitude (black lines in Figure 4b and d). This capability to fine-tune the wave localization length can be extended to other incident angles, even allowing for precise control across multiple angles (Supplementary Note S4). Notably, when considering *L* = 2*λ* and *n*_EMT_ = 2.648, the overall structures lie in the regime of the broken Born approximation. The results in Figures 3 and 4 confirm that *S*(*k*) serves as an excellent design tool for engineering disorder in deep-subwavelength microstructures, even when the entire system exhibits strong scattering. When considering the scaling theory of light [44], which states the ultimate wave localization in 1D disordered structures, our approach corresponds to a method of tailoring the degree of localization based on deep-subwavelength-scale engineering.

## 3 Discussion

Achieving optical functionalities with engineered disorder in deep-subwavelength scales transforms traditional design strategies in two key aspects: designing microstructure correlations in subwavelength scales and multiple scattering regimes. First, while tailoring spatial [1], [41], [45], [46], [47], [48], [49] or temporal [50], [51], [52] correlations in space-time material microstructures has been widely investigated, angular optical functionalities achieved in this work reveal how wave behaviours at the GH regime can lower the length-scale boundary in microstructure correlations that affect wave phenomena substantially. Second, although our structures should be treated as multiple scattering structures, as evidenced by the multilayer thicknesses (*L* = 2*λ*–6*λ*), effective index (*n*_EMT_ = 2.648), and notably low transparency (or small *ξ*), the structure factor *S*(*k*) enables the deterministic design of optical functionalities at deep-subwavelength scales. Both aspects emphasize the necessity of extending the concept of disordered photonics to subwavelength optics.

One intriguing future study related to our results would be the design of photonic bandgap (PBG) materials using deep-subwavelength disordered structures. Notably, there have been significant achievements in realizing and examining PBG under the concept of SHU materials [1], [2], [3], [53], [54], [55], [56], [57] to obtain isotropic bandgaps. In these studies, the phase evolutions necessary for constructing PBG originate from light propagations along the bulk of materials. In this context, the utilization of phase evolutions at the interfaces for manipulating light scattering provides extended design freedom for PBG realizations at deep-subwavelength scales. For this future research direction, the design of higher-dimensional metamaterials and metasurfaces for the broad-angle GH shift will be necessary.

Although our study exemplified a simple structure – two-phase multilayers – the underlying concept is applicable to other physical domains: spectral responses, higher-dimensional systems, open systems, time-varying photonics, and quantum optics. For instance, the proposed design directly enables spectral control of localization through the definition of structure factors, thereby inspiring the design of color selectivity at deep-subwavelength scales. We can also envision implementing two- or three-dimensional SHU systems and their deformations using deep-subwavelength elements, such as quantum dots [28]. Although we focused on Hermitian systems with reciprocal structure factors in our work, the extension to open systems allows for imposing directionality on scattering. Additionally, for GHz or THz systems, subwavelength temporal crystals and disorder can be introduced through nonlinear optics [50], [58]. The recently demonstrated temporal Goos–Hänchen shift can be extended to these temporal systems [59]. Engineering in deep-subwavelength scales also inspires the connection to quantum phenomena, which will provide unique methodologies in manipulating quantum-mechanical quantities of photons [60], [61].

In conclusion, we demonstrated angle-selective engineering of localization in deep-subwavelength disordered multilayers. By tailoring the profile of the structure factor *S*(*k*), we achieved the inverse design of deep-subwavelength crystals, SHU, and Poisson uncorrelated disorder, as well as nontrivial transitions between these microstructural phases. We identified unique localization properties associated with each microstructural phase under the breakdown of EMT, especially near the regime of pronounced Goos–Hänchen effects at the layer interfaces. The independent manipulation of long-range and short-range order enables highly selective control of wave localization. The result paves the way for designing deep-subwavelength optical structures within the context of disorder engineering, thereby enhancing the versatility and functionalities of disordered metamaterials.

## 4 Methods

### 4.1 Deep-subwavelength multilayers

The refractive indices of multilayers are assumed to be *n*_L_ = 1.03 for silica aerogel layers and *n*_H_ = 3.6 for gallium phosphide (GaP) layers. The multilayers are surrounded by a homogeneous medium composed of silicon with *n*_ext_ = 4.3. We assume the lossless condition for these materials.

### 4.2 Inverse design process

To achieve the target profile of *S*(*k*) in the designed material, we employ the following iterative optimization process. First, we determine the parameters for a crystal and calculate its *S*(*k*) (Figures 1a and 2a) by following Supplementary Note S1. We also set the target structure factor *S*_0_(*k*) for the microstructural phase of interest (Figure 1b–e). Second, we obtain an initial state by perturbing 10^4^ randomly sampled *n*_H_ layers with replacement to enhance the convergence of the optimization method. The perturbation in preparing the initial state is uniformly random and its statistical range is set to be maximized while ensuring the hard particle condition of *n*_H_ layers. We then apply 2 × 10^5^ iterative optimization processes to the initial state.

In each iteration, we utilize the cost function defined by the mean squared error (MSE). To enhance the convergence performance, we calculate the weighted MSE for each discretized range of the reciprocal axis *k*. First, for the reciprocal space of interest *K*_I_, we divide *K*_I_ into the subspaces *κ*_*j*_ (*j* = 1, 2, 3, and 4). The MSE for the *j*th subspace is defined as follows:

(2) $$\text{MS}{\text{E}}_{j}=\frac{1}{\left|\kappa_{j}\right|}\underset{k\in \kappa_{j}}{\int }{\left(\frac{S\left(k\right)-S_{0}\left(k\right)}{S_{0}\left(k\right)}\right)}^{2}\text{d}k,$$

where |*κ*_*j*_| is the length of the subspace *κ*_*j*_. Second, to reflect the distinct importance of each subspace in the structure factor, the total cost function is defined as:

(3) $$\text{MS}{\text{E}}_{\text{total}}=\frac{\overset{4}{\underset{j=1}{\sum }}w_{j}\text{MS}{\text{E}}_{j}}{\overset{4}{\underset{j=1}{\sum }}w_{j}},$$

where the values of the weighting factor *w*_*j*_ are determined empirically for each microstructural phase. Finally, with the designed cost function in Eq. (3), we apply the uniformly random perturbation to *n*_H_ layers with the maximum statistical range within the hard particle condition. To hinder the local minima issue, we apply the modified Metropolis acceptance rule [38] of which the acceptance probability is determined by empirical hyperparameters according to simulated annealing method [33]. The proposed optimization process shows an excellent performance as shown in Figures 2–4. An example of the optimization process is described in Supplementary Note S2.

## Supplementary Material

Supplementary Material Details
