# Supplementary material for: Deep-subwavelength engineering of stealthy hyperuniformity
Source: Nanophotonics. 2025 Jan 7;14(8):1113–22. doi: 10.1515/nanoph-2024-0541 (PMC12019948; doi:10.1515/nanoph-2024-0541)
Supplement: Supplementary file 1 — Supplementary Material Details [file j_nanoph-2024-0541_suppl_001.pdf]

Supplementary Information for "Deep-subwavelength engineering of stealthy hyperuniformity"

Jusung Park[1,2†], Seungkyun Park[1,2†], Kyuho Kim[2], Jeonghun Kwak[3], Sunkyu Yu[2*], and Namkyoo Park[1*]

[1]Photonic Systems Laboratory, Department of Electrical and Computer Engineering, Seoul National University, Seoul 08826, Korea

[2]Intelligent Wave Systems Laboratory, Department of Electrical and Computer Engineering, Seoul National University, Seoul 08826, Korea

[3]Department of Electrical and Computer Engineering, Inter-university Semiconductor Research Center, and SOFT Foundry Institute, Seoul National University, 1, Gwanak-ro, Gwanak-gu, Seoul 08826, Korea

[†]These authors contributed equally to this work.

E-mail address for correspondence: *sunkyu.yu@snu.ac.kr, *nkpark@snu.ac.kr

Note S1. Structure factor calculation

Note S2. Iterative optimization process

Note S3. Examples of disordered multilayer patterns

Note S4. Localization length with multiple angular selectivity

Note S1. Structure factor calculation

The structure factor $S(k)$ in a one-dimensional (1D) inhomogeneous material of length L for the refractive index profile $n(z)$ is defined as follows:

$$S(k) = \frac{1}{L}\left|\int_0^L n^2(z)e^{-ikz}dz\right|^2. \tag{S1}$$

To analyse the two-phase material composed of the binary-value refractive indices n_{H} and n_{L}, we model the material as a series of the n_{H} layer embedded in the background material of n_{L} (Fig. S1). The Fourier transform of $n^2(z)$ then becomes:

$$\begin{aligned}
F\left\{n^2(z)\right\} &= \int_{-\infty}^{\infty} n^2(z)e^{-ikz}dz \\
&= (n_{\mathrm{H}}^2 - n_{\mathrm{L}}^2)F\left\{\sum_{j=1}^{l}\delta(z - z_{\mathrm{H},j}) * \mathrm{rect}\left(\frac{z}{d_{\mathrm{H},j}}\right)\right\} + F\left\{n_{\mathrm{L}}^2\right\} \\
&= (n_{\mathrm{H}}^2 - n_{\mathrm{L}}^2)\left(\sum_{j=1}^{l} F\left\{\delta(z - z_{\mathrm{H},j})\right\}F\left\{\mathrm{rect}\left(\frac{z}{d_{\mathrm{H},j}}\right)\right\}\right) + F\left\{n_{\mathrm{L}}^2\right\} \\
&= (n_{\mathrm{H}}^2 - n_{\mathrm{L}}^2)\left(\sum_{j=1}^{l} e^{-ikz_{\mathrm{H},j}} d_{\mathrm{H},j}\,\mathrm{sinc}\frac{kd_{\mathrm{H},j}}{2\pi}\right) + n_{\mathrm{L}}^2\delta(k=0),
\end{aligned} \tag{S2}$$

where F denotes the Fourier transform, $\mathrm{rect}(z/d_{\mathrm{H},j})$ is rectangular function of the jth n_{H} layer with the width $d_{\mathrm{H},j}$, the symbol '$*$' denotes the convolution, δ represents the Dirac delta function, $z_{\mathrm{H},j}$ is the center of the jth rectangular function, and l is the number of the n_{H} layers. Considering the thermodynamic limit of $L\to\infty$, the structure factor for the finite-length material is calculated as follows:

$$S(k) = \frac{1}{L}\left|(n_{\mathrm{H}}^2 - n_{\mathrm{L}}^2)\left(\sum_{j=1}^{l} e^{-ik\cdot z_{\mathrm{H},j}} d_{\mathrm{H},l}\,\mathrm{sinc}\frac{kd_{\mathrm{H},l}}{2\pi}\right) + n_{\mathrm{L}}^2\delta(k=0)\right|^2. \tag{S3}$$

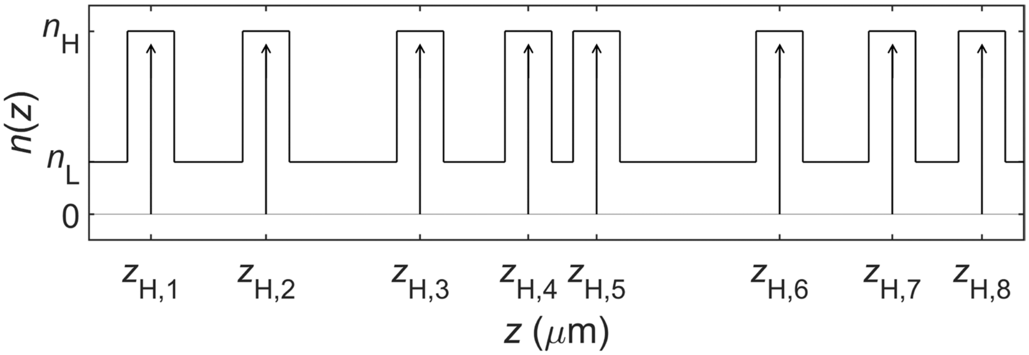

Fig. S1. Refractive index profile for two-phase materials. The optical potential is described by the refractive index profile $n(z)$, which is composed of the rectangular functions that describe the n_H layers.

Note S2. Iterative optimization process

Figure S2 describes an example of the optimization process for the structure factor of 1D SHU in Figure 2b in the main text. While Fig. 2a illustrates the decrease of the cost function defined by the MSE (see Methods), Fig. 2b shows an excellent agreement between the target structure factor and the obtained structure factors. Notably, the design process can also be considered including the randomly perturbed Fourier transforms, as described in Supplementary Note S1.

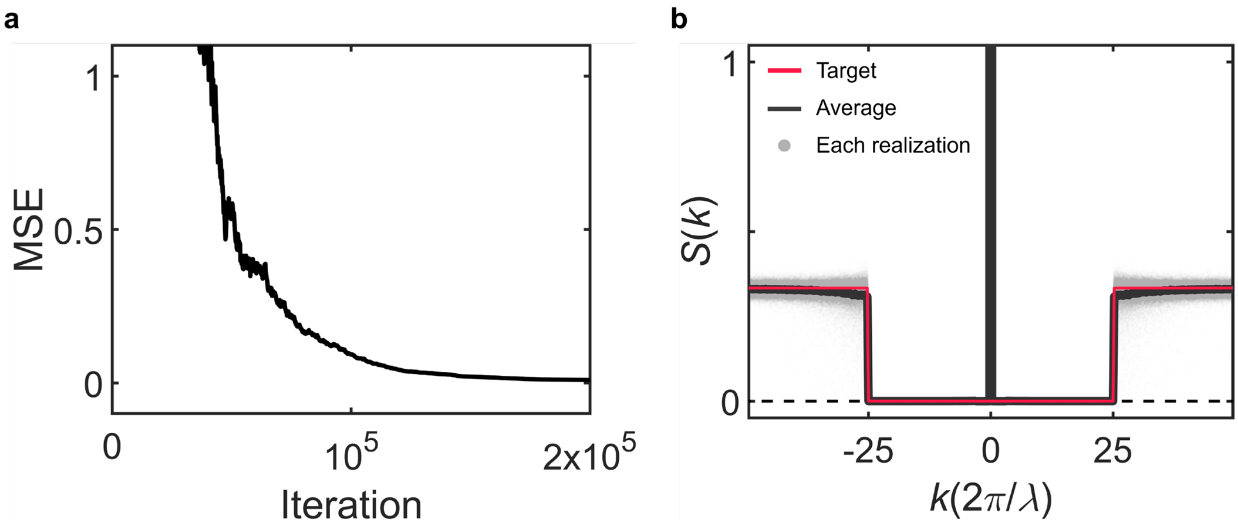

Fig. S2. *S*(*k*) design example. a, The evolution of the MSE during the iterative optimization processes. **b,** The target (red line) and the obtained (black points for each realization and black line for the average) structure factor. $L = 4\lambda$. An ensemble of 10^3 realizations is examined.

Note S3. Examples of disordered multilayer patterns

Figure S3 present examples of disordered material patterns at each phase of Fig. 3 in the main text. For visibility, we show a part (100 nm length) of each sample (1 µm).

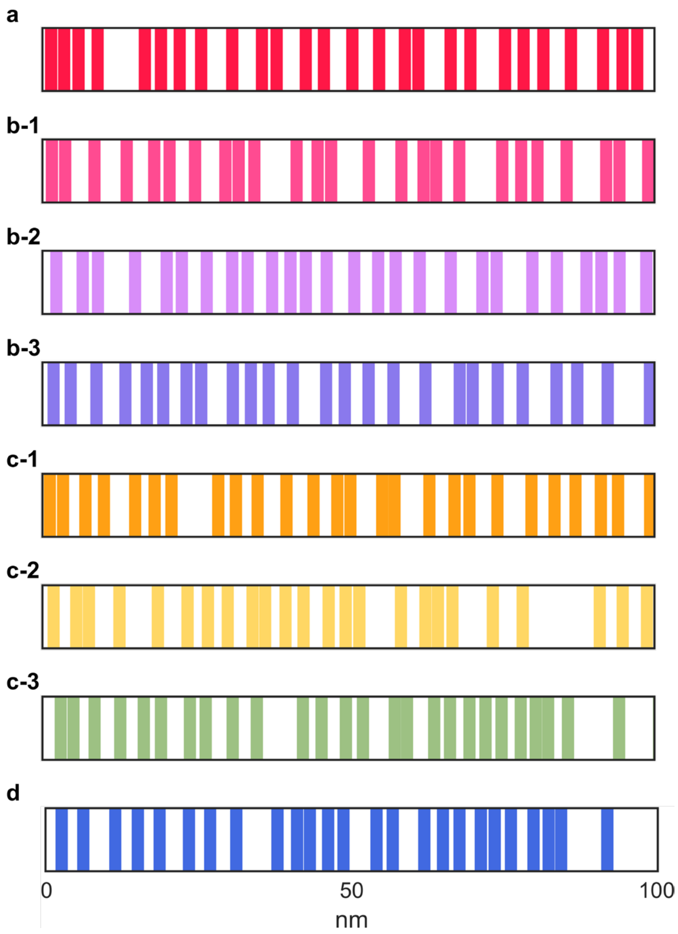

Fig. S3. Examples of disordered multi-layered media for the two cases of material phase transitions. a-d, Schematic representation of refractive index profiles: SHU (**a**), breakdown of long-range order (**b: b-1, b-2, and b-3**), decrease of K (**c: c-1, c-2, and c-3**), and uncorrelated disorder (**d**), corresponding to Fig. 3 in the main text. White and coloured regions correspond to n_L (low-index material) and n_H (high-index material) refractive indices, respectively.

Note S4. Multiple angle selectivity

By controlling $S(k)$, we can manipulate the localization length for multiple incident angles (Fig. S4). Starting from the SHU peaks at $K = 11\pi/\lambda$, we adjust the $S(k)$ at $|k| = 9\pi/\lambda$ and $7\pi/\lambda$ (Fig. S4a,c). The corresponding incident angles of the controlled localization length are $\theta = 24°$ and $31°$ (Fig. S4b,d). The increase and decrease of the target length-scale density fluctuations lead to the suppression (Fig. S4b) and enhancement (Fig. S4d) of the localization length, respectively, demonstrating the ability to realize multiple-angle selectivity in controlling wave localization.

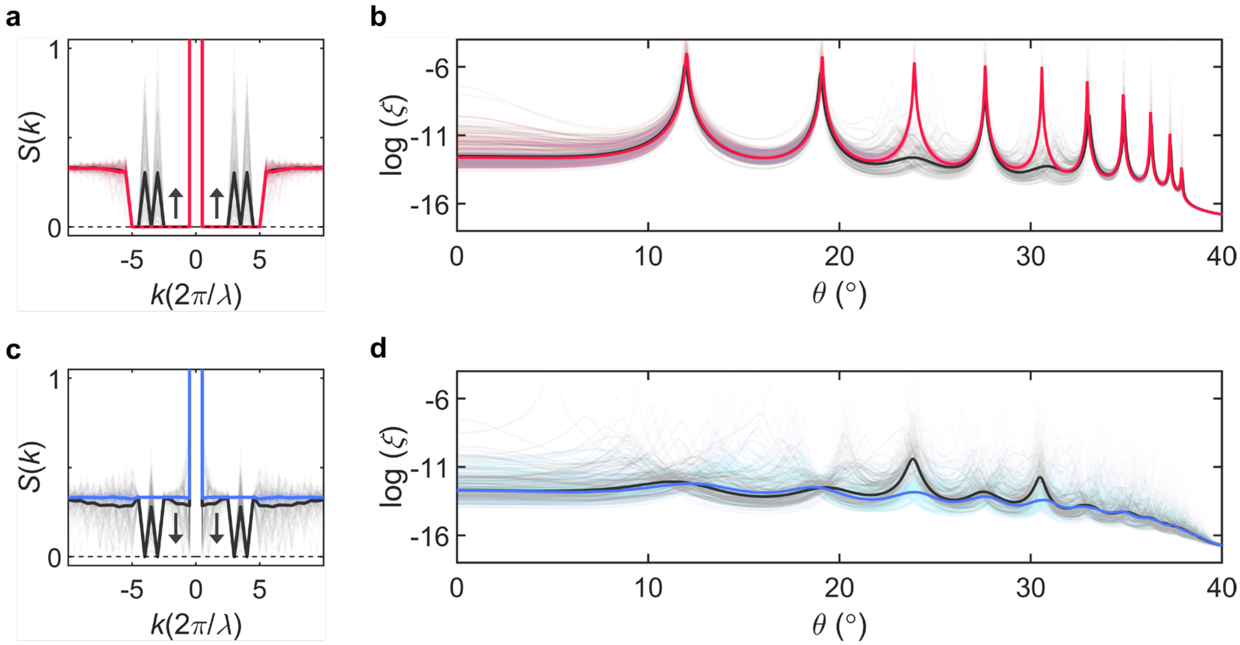

Fig. S4. Multi-angle selective localization. a,b, Selective annihilation and **c,d,** selective creation of the target Fabry-Perot resonances: the designed $S(k)$ transitions (**a,c**) and incident-angle-dependent localization lengths (**b,d**). In **a,c**, the black arrows illustrate the transition for each case. All the other parameters and plots are the same as those in Fig. 3 in the main text.
